# The predictive role of parental attitudes toward COVID-19 vaccines and child vulnerability: A multi-country study on the relationship between parental vaccine hesitancy and financial well-being

**DOI:** 10.3389/fpubh.2023.1085197

**Published:** 2023-02-16

**Authors:** Hamid Sharif Nia, Kelly-Ann Allen, Gökmen Arslan, Harpaljit Kaur, Long She, Fatemeh Khoshnavay Fomani, Ozkan Gorgulu, Erika Sivarajan Froelicher

**Affiliations:** ^1^Traditional and Complementary Medicine Research Center, Addiction Institute, Mazandaran University of Medical Sciences, Sari, Iran; ^2^School of Educational Psychology and Counselling, Faculty of Education, Monash University, Melbourne, VIC, Australia; ^3^Department of Psychological Counselling and Guidance, Mehmet Akif Ersoy University, Burdur, Türkiye; ^4^Centre for Wellbeing Science, University of Melbourne, Melbourne, VIC, Australia; ^5^School of Management and Marketing, Faculty of Business and Law, Taylor's University, Subang Jaya, Malaysia; ^6^Faculty of Business, Design and Arts, Swinburne University of Technology, Kuching, Sarawak, Malaysia; ^7^School of Nursing and Midwifery, Tehran University of Medical Sciences, Tehran, Iran; ^8^Department of Biostatistics and Medical Informatics, Kirşehir Ahi Evran University Faculty of Medicine, Kırşehir, Türkiye; ^9^Department of Physiological Nursing, School of Nursing, University of California, San Francisco, San Francisco, CA, United States; ^10^Department of Epidemiology and Biostatistics, School of Medicine, University of California, San Francisco, San Francisco, CA, United States

**Keywords:** parental attitudes toward COVID-19 vaccines, child vulnerability, parental vaccine hesitancy, financial well-being, mediation study

## Abstract

**Background and purpose:**

Recent new mutations and increases in transmission of COVID-19 among adolescents and children highlight the importance of identifying which factors influence parental decisions regarding vaccinating their children. The current study aims to explore whether child vulnerability and parents' attitudes toward vaccines mediate the association between perceived financial well-being and vaccine hesitancy among parents.

**Method:**

A predictive, cross-sectional, multi-country online questionnaire was administered with a convenience sample of 6,073 parents (Australia, 2,734; Iran, 2,447; China, 523; Turkey, 369). Participants completed the Parent Attitude About Child Vaccines (PACV), the Child Vulnerability Scale (CVS), a Financial Well-being (FWB) measure, and Parental Vaccine Hesitancy (PVH) questionnaire.

**Results:**

The current study revealed that perceived financial well-being had significant and negative associations with parents' attitudes toward COVID-19 vaccines and child vulnerability among the Australian sample. Contrary to the Australian findings, results from Chinese participants indicated that financial well-being had significant and positive predictive effects on parent attitudes toward vaccines, child vulnerability, and parental vaccine hesitancy. The results of the Iranian sample revealed that parents' attitudes toward vaccines and child vulnerability significantly and negatively predicted parental vaccine hesitancy.

**Conclusion:**

The current study revealed that a parents' perceived financial well-being had a significant and negative relationship with parental attitudes about vaccines and child vulnerability; however, it did not significantly predict parental vaccine hesitancy among Turkish parents as it did for parents in Australia, Iran, and China. Findings of the study have policy implications for how certain countries may tailor their vaccine-related health messages to parents with low financial wellbeing and parents with vulnerable children.

## Introduction

Nowadays, some countries are preparing to announce the end of the COVID-19 pandemic. However, immunization remains a unique measure for protecting the population against this disease. The increasing number of COVID-19 cases and deaths has led governments worldwide to launch preventive strategies to control the pandemic (1–3) which assisted in flattening the pandemic curve, but there has been a resurgence in cases reported since the economies reopened (4, 5) and new variants emerged (6, 7). One of the strategies to curb the spread of the disease is the development of the COVID-19 vaccines which stimulate the immune system to produce antibodies against the virus (8). Promoting vaccination is crucial, especially among children and adolescents. However, due to the unprecedented speed and scale the vaccines were developed in some countries and settings (1, 9, 10), concerns regarding its effectiveness and safety have emerged (11–13), prompting vaccine hesitancy among healthcare workers (9, 14, 15), parents (16, 17), university students (18, 19), expectant mothers (20), and the general public (21–23).

Vaccine hesitancy is defined as the “delay in acceptance or refusal of vaccination despite availability of vaccination services” (24); p. 4,163. Previous research has linked vaccine hesitancy to age (9, 14, 25), education (26), occupation (9, 14, 27), trust (28–30), religious practices and beliefs (31, 32), vaccine misinformation (33–35), social media (36), miscalculation of risk and lack of knowledge (37), and gender (25, 38). Even though the number of cases of COVID-19 is increasing, vaccine hesitancy seems to be high across countries ranging from 10 to 50% (New Zealand 30%, Portugal 65%, Japan 43.9%, US 22%, and Singapore 33%) (39–43). The lowest rates of vaccine acceptance have been reported in the Middle East, Russia, Africa and several European countries (44).

One of the most significant public health challenges globally is addressing parental vaccine hesitancy, which has been identified previously for polio immunizations (45), measles, mumps and rubella (MMR) (46, 47), routine childhood vaccinations (48), and now for COVID-19 vaccines (26, 49). The acceptance of vaccination for children is highly influenced by parents' attitudes and feelings over the decision to vaccinate which varies from total acceptance to complete refusal (50, 51). Parents often worry about a combination of potential side effects of the vaccine (52–54), fear of compromising their children's immune systems (55), religious beliefs (56), and the fear of autism (57). This is linked to a lack of trust in the government (58, 59), the pharmaceutical industries (60, 61), and health providers (62, 63) as many presume there are motives behind promoting vaccinations (63, 64). These are often associated with newer vaccines (17) or the dissemination of vaccine misinformation from health care providers (59, 65) and the media (66, 67). Interestingly, studies have indicated that fathers, parents who are not vaccinated (68, 69), and negative vaccination experiences (70) play major roles in parents being hesitant about vaccinating their children. Resolving doubts on vaccination to provide higher immunization coverage for children is a critical concern for policymakers. There is a consensus that safe COVID-19 vaccines can end the current pandemic, and vaccine acceptance is as crucial as vaccine safety and effectiveness in the successful pandemic control (44).

## Vaccine hesitancy

While vaccine hesitancy can present the individuals' adherence and acceptance of recommended vaccines for themselves (71), parental vaccine hesitancy may indicate that parents accept to vaccinate their children but are concerned about the vaccine's efficacy and safety (72). Vaccine-hesitant individuals may agree to some vaccines while refusing the other recommended vaccine for themselves or their children (73). Several individual, social, and cultural factors can determine the individual and parental willingness to get vaccines for themselves or their children (72). Although personal vaccine hesitancy does not necessarily lead to parental vaccine hesitancy, existing knowledge suggests a significant positive correlation between these two variables (74). Vaccine hesitancy has been identified as a public health challenge during the COVID-19 pandemic. The common personal reasons for refusing COVID-19 vaccines include concerns about safety and effectiveness, as well as the lack of trust in the vaccine's origin. People believe that the vaccines produced in a rush may be very dangerous or useless against COVID-19 (75). Similar to the other vaccines, parental hesitancy to the COVID-19 vaccines is a global public health concern and many studies focused on the factors that determine parents' willingness and intention to vaccinate their children against COVID-19 (76–78). Parental vaccine hesitancy hinders the immunization efforts for children against COVID-19 that aim to protect their health, as well as that of their community. Although some factors such as trust, attitude toward the COVID-19 vaccine safety and effectiveness, the perceived COVID-19 risk by parents, and parental satisfaction with social relations have been identified to be correlated with parental COVID-19 vaccine hesitancy (74, 79), further studies are recommended to investigate the factors affecting parental COVID-19 vaccine hesitancy.

## Child vulnerability

Parental hesitancy over vaccination is closely linked to child vulnerability. Child vulnerability is defined as a parent's belief that a child is vulnerable to developmental or behavioral problems, and illness, or death (80). Considering Green and Solnit (81) and Forsyth et al. (80) suggest two underlying concepts that determine parents' perception of their child's vulnerability. These two concepts include the instance in which the child is medically vulnerable because of an existing health condition and the second concept include the instance in which the parents fear that their child may die (80). Past studies have found inconclusive relationship between vaccine hesitancy and child vulnerability variables (82, 83). However, parents of children with asthma, obesity, and other comorbid conditions were more hesitant in vaccinating their children (16) due to the risk of infection and vaccine side effects (70). Some parents who refuse vaccination for their children feel that their children are healthy and less vulnerable to the disease (70). On the contrary, with the increasing awareness on the need for immunization as COVID-19 severity increases and affects the health of vulnerable children, some parents approve vaccination (16).

## Perceived financial well-being

Perceived financial well-being or the perception that an individual can fully meet their financial obligations now and in the future, has been found to negatively impact the hesitancy of parents toward vaccination (54, 83, 84). Parents who have low financial well-being tend to have diminished access to healthcare (80) and they are concerned about the cost of vaccinations, or potential medical costs if child experience an adverse reaction. Some studies suggest that parents experiencing financial pressures and stress are more likely to question the necessity and safety of vaccines than parents who have fewer financial concerns and high financial well-being (85). Generally, existing literature indicated that both low (85) and high financial status (86) can be considered as determinant factors of vaccine hesitancy among parents. Vaccination has been highly effective at decreasing the spread of some communicable diseases (87), thus mitigating childhood morbidity and mortality (88). With the new mutations and increased transmission of COVID-19 among young populations (89), it is important for this age group to be vaccinated to prevent further viral spread. In addition, to ensure that the vaccination efforts are at satisfactory levels, there is a need to overcome barriers related to parents' perceptions of low financial well-being and child vulnerability. Considering that research on this area is limited, the current study aims to investigate the relationships between financial well-being and vaccine hesitancy among parents by determining the drivers of hesitancy (83, 90). This study also aims to further explore whether child vulnerability and parent attitudes about vaccines mediate the association between financial well-being and vaccine hesitancy among parents.

## Conceptual framework

Based on Roger's protection motivation theory (PMT) (1975, 1983), it is hypothesized that parental vaccine hesitancy is shaped by financial well-being (FWB), parent attitude about child vaccines (PACV), and child vulnerability (CVS). This theory justifies one's motivation to participate in protective behaviors, which are encouraged by threat stimulus (91). Based on PMT, a parent's decision of whether to participate in protective behaviors depends on two cognitive processes: coping and threat appraisal (92). Threat appraisal refers to one's adaptive actions which consist of threat severity, maladaptive rewards, and threat vulnerability (93) whereas coping appraisal indicates the ability of the individual to engage in protective behaviors in the presence of threat, (94) whereas threat appraisal refers to one's adaptive actions consist of threat severity, maladaptive rewards, and threat vulnerability. The uncertainties surrounding the side effects of the COVID-19 vaccine in children have caused increasing levels of fear among parents and may motivate parents to adopt protective behaviors as they may feel that there is no definitive treatment for the disease (95). The fear is further exacerbated by the vulnerability of the children, thus increasing the hesitancy of the vaccine among parents. In order to improve protection motivation, identifying and addressing the causes of reluctance through coping and threat appraisal procedures are needed.

## Study hypotheses

In the light of the proposed conceptual research model and literature, the following hypotheses were developed:

Hypothesis 1: Financial well-being (FWB) is positively related to parent attitude about child vaccines (PACV).
Hypothesis 2: Financial well-being (FWB) is negatively related to child vulnerability (CVS).
Hypothesis 3: Financial well-being (FWB) is negatively related to parental vaccine hesitancy (PVH).
Hypothesis 4: Parent attitude about child vaccines (PACV) is negatively related to parental vaccine hesitancy (PVH).
Hypothesis 5: Child vulnerability (CVS) is related to parental vaccine hesitancy (PVH).
Hypothesis 6: Parent attitude about child vaccines (PACV) and child vulnerability (CVS) mediate the negative relationship between financial well-being (FWB) and parental vaccine hesitancy (PVH).

## Method

### Study design and participants

**Figure 1:**
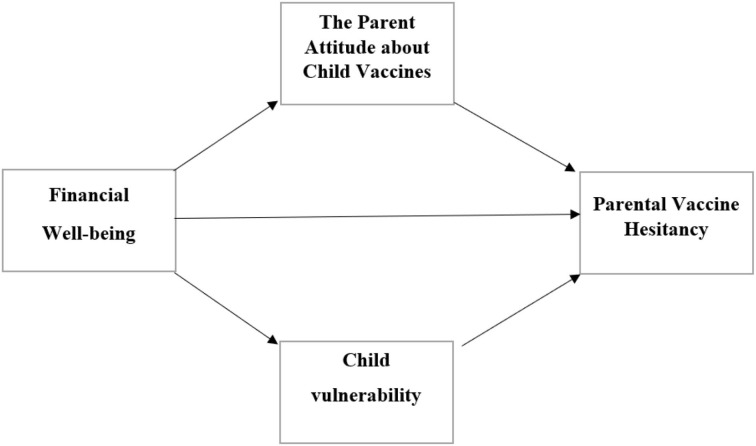
The proposed mediating model.

A cross-sectional, multi-country online study design was used to investigate the relationships between perceived financial wellbeing and parental vaccine hesitancy due to the pandemic, as well as the mediating role of the parents' attitudes toward COVID-19 vaccines and child vulnerability in these relationships (Figure 1). Data were collected over eight weeks, between 8 August 2021 and 1 October 2021. Parental vaccine hesitancy can be determined by a variety of individual and social factors identified in different study settings (44, 96). In this study, data were gathered from Australia, China, Turkey, and Iran which have almost similar COVID-19 vaccine hesitancy ranges (between 30 and 45%) among the general population despite their different socio-economic status (97–100).

Inclusion criteria were: being a parent/caregiver of at least one child, having accessibility to a smartphone or another digital device to answer the web-based questionnaire, and having the ability to read the questionnaire items. The participants were recruited from Australia, Iran, China, and Turkey.

Data were gathered using a convenience sampling method along with probability sampling to reduce bias. The online questionnaire was prepared using Google form and a cover letter was included to provide the research aims and relevant information about the study. The questionnaire link was shared via popular messaging apps (e.g., WhatsApp, LinkedIn, Instagram, and Telegram) and in different virtual groups such as work groups, scientific groups, the newsgroup, and other popular groups. The questionnaire cover letter was prepared to introduce the research aims and provide any related information regarding the study. A total of 6,073 parents (Australia, 2,734; Iran, 2,447; China, 523; Turkey, 369) filled out the online questionnaire. The Iranian data were gathered in two phases: 1,187 parents participated in the study during the pre-5th waves of the COVID-19 outbreak, and 1,260 respondents contributed during the post-5th waves. The study participants' characteristics are presented in Table 1.

**Table 1:** Demographic characteristics of respondents and mean (SD) of COVID-19 vaccine hesitancy among parents.

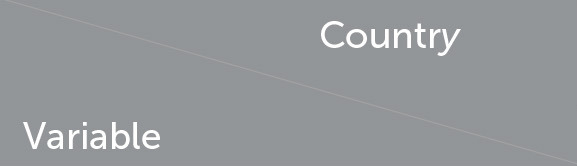		**Australia** **(*****n*** **=** **2734)**		**China** **(*****n***** =** **523)**		**Iran** **(*****n***** =** **2447)**		**Turkey** **(*****n***** =** **369)**	
**Variable**		***n*** **(%)**	***M*** **(sd)**	***n*** **(%)**	***M*** **(sd)**	***n*** **(%)**	***M*** **(sd)**	***n*** **(%)**	***M*** **(sd)**
Parents' Gender	Female Male Other	2611 (95.5) 116(4.2) 6 (0.2)	34.7 (6.1) 33.4 (6.5) 37.7 (4.3)	340 (65) 183 (35) 0 (0)	21.5 (4.2) 21.8 (5.2) 0 (0)	1990 (81.32) 433 (17.69) 24 (0.98)	36.1 (4.2) 35.3 (5.2) 34.4 (0)	170 (46.1) 199(53.9) 0 (0)	34.0 (5.3) 34.1 (4.2) 0 (0)
			*P =* 0.064, F = 2.7		*P =* 0.547, F = 0.3		*P =* 0.001, F = 7.2		*P =* 0.718, F = 0.1	
Parents' Age	< 20 years old 20–40 years old 40–60 years old 60 and more	3 (0.1) 1,208 (44.2) 1,517 (55.5) 6 (0.2)	33.6 (6.6) 34.4 (6.3) 34.9 (6.0) 23.5 (7.5)	12(2.3) 483 (92.3) 28 (5.4) 0 (0)	19.6 (9.7) 21.6 (4.4) 22.6 (4.6) –	24 (0.98) 1,508 (61.62) 904 (36.95) 11 (0.45)	35.8 (5.4) 35.9 (4.1) 35.9 (4.2) 37.0 (2.7)	0 (0) 118 (32) 251 (68) 0 (0)	– 34.0 (4.9) 34.1 (4.7) –
			*P < * 0.001, F = 8.1		*P =* 0.165, F = 1.8		*P =* 0.800, F = 0.3		*P =* 0.880, F = 0.0	
Child vaccination history	Yes^*^ No^*^	2515 (92) 219 (8)	35.8 (4.6) 21.1 (5.9)	335 (64.1) 188 (35.9)	21.3 (4.4) 22.1 (4.8)	1983 (81.03) 464 (18.9)	36.2 (4.01) 34.6 (4.6)	286 (77.5) 83 (22.5)	34.3 (4.7) 33.3 (4.9)
			*P < * 0.001, t = 35.9		*P =* 0.061, t = −1.8		*P < * 0.001, t = 6.6		*P =* 0.112, t = 1.5	
Child Chronic disease or abnormality	Positive Negative	313 (11.4) 2,421 (88.6)	35.2 (6.1) 34.6 (6.2)	75 (14.3) 448 (85.7)	22.1(5.8) 21.5 (4.3)	276 (11.3) 2,171 (88.7)	36.2 (4.4) 35.9 (4.1)	60 (16.3) 309 (87.3)	33.4 (5.4) 34.2 (4.6)
			*P =* 0.071, t = 1.8		*P =* 0.231, t = 1.0		*P =* 0.235, t = 1.1		*P =* 0.278, t = −1.0	
Child COVID-19 history	Positive Negative The parent is not sure	33 (1.2) 2,627 (96.1) 74 (2.7)	31.9(8.0) 34.9 (5.9) 26.2 (8.5)	26 (5) 476 (91) 21 (4)	19.8 (6.4) 21.5 (4.2) 27.1 (5.5)	838 (34.24) 1,379 (56.35) 230 (9.40)	35.7 (4.3) 36.0 (4.1) 36.1 (4.0)	78(21.1) 278 (75.3) 13 (3.5)	34.4 (4.2) 33.9 (5.0) 35.6 (2.7)
			*P < * 0.001, F = 78.0		*P < * 0.001, F = 18.2		*P =* 0.230, F = 1.4		*P =* 0.307, F = 1.1	

* Yes: The child is up-to-date with the vaccination schedule, No: The child is not up-to-date with the vaccination schedule.

### Measurements

#### Sociodemographic characteristics

The respondents' sociodemographic variables which included age, gender, level of education, living area, child vaccination history, and child COVID-19 history were collected.

#### Translation procedures

The survey questionnaire contained sociodemographic information, Parent Attitudes about Child Vaccines (PACV), the Child Vulnerability Scale (CVS), Financial Well-being (FWB), and Parental Vaccine Hesitancy (PVH). Beaton et al.'s (101) instruction was used for translation and back-translation procedure. For countries where the first language was not English, all questionnaires were translated into the languages of the countries (Chinese, Persian, and Turkish). All translators were bilingual individuals. Two translators independently translated the questionnaires into the study setting language. The research team then assessed the translated versions and selected the best item translation. Following this step, two other bilingual translators who were “blinded” to the original version of the questionnaire conducted the back-translation procedure independently. The expert committee (consisting of research team members, two nurses, one physician in social medicine, and a methodologist) then checked the back-translated version to ensure the accuracy and equivalence between it and the original questionnaire version. Also, the committee assessed the cross-cultural equivalence and appropriateness of the questionnaire to the study population, as well as the semantic equivalence of the items. No item was changed during the procedure.

#### The parent attitude about child vaccines (PACV)

To investigate parental perceptions of vaccine safety, the sub-scale of safety and efficiency of the PACV questionnaire was used. The questionnaire consists of 15-item, 3-factor measures (two items on vaccine behavior, four items on beliefs about vaccine safety and efficiency, and nine items on general attitudes). A five-points Likert-scale questionnaire ranging from “strongly agree (5 scores)” to “strongly disagree (1 score)” was used to gather the data. Higher scores indicate more negative attitudes toward the vaccine (102).

#### The child vulnerability scale (CVS)

In order to investigate the parental perception of child vulnerability. The CVS is an 8-item self-report measure with 2 factors including “child medical condition” and “prior fears that child might die”. Participants were asked to rate their perception on their child's vulnerability using a five-points Likert-scale questionnaire ranging from “strongly agree (5 scores)” to “strongly disagree (1 score).” Higher scores indicated more perceived vulnerability (80).

#### Financial well-being (FWB)

Perceived financial well-being was measured by five items adapted from the CFPB's Financial Well-Being Scale (103). The CFPB's scale included the concepts of “financial situation” and “capability” and uses a 5-point Likert scale from “strongly agree (1 scores)” to “strongly disagree (5 score)”. Higher scores indicated more perceived financial well-being. A reverse scoring was used for item number 4 (I have money left over at the end of the month)”.

#### Parental vaccine hesitancy (PVH)

A 10-item, 2-factor measure consisting of “lack of confidence” and “risk” categories was used. The scale is measured on a five-point Likert-type rating scale ranging from “strongly disagree (1 score)” to “strongly agree (5 scores)”. Higher scores indicate more hesitancy (104).

### Ethical consideration

The Ethics Committee of Mazandaran University of Medical Sciences, Iran approved the Ethical Considerations of this study (Reference No: IR.MAZUMS.REC.1400.189). In addition, all participants were informed of the purpose of the data collection, and questionnaires were distributed to the respondents only after they provided their consent to participate in the survey. Moreover, the respondents were ensured that their participation was on a voluntary basis and the confidentiality of all collected data was guaranteed.

### Data analyses

A series of path analyses were used to explore the direct and indirect associations between parent attitudes about vaccines, child vulnerability, financial well-being, and parental vaccine hesitancy. Observed scale characteristics for all samples in the study were first examined. As recommended by Hair et al. (105), skewness and kurtosis scores were utilized to evaluate the assumption of normality for the study variables. Pearson correlation analysis was then performed to examine the relationships between the variables. In addition, the reliability of the measures was examined utilizing internal reliability (α) estimates. Finally, structural equation modeling was conducted to test the mediating role of parent attitudes about vaccines and child vulnerability in the link between financial well-being and parental vaccine hesitancy. Some model fit statistics, with their decision points, were examined to interoperate the results of path models: comparative fit index (CFI) and Tucker-Lewis index (TLI) scores ≥0.90 = an adequate data–model fit; and the root mean square error of approximation scores (RMSEA; with 90% confidence interval) ≤ 0.10 = an acceptable model fit (106). Before testing the mediation analyses, a series of measurement models were also carried out. Similar to structural modeling, data-model fit statistics were used to evaluate the results of this analysis. Additionally, multiple group analyses were performed to compare the direct and indirect associations between the variables of the study in the samples from different cultures. All study analyses were conducted utilizing SPSS v25 and AMOS v24.

## Results

Observed scale characteristics results are presented in Table 2. Skewness and kurtosis scores were at an acceptable range suggesting that all measures in the study had relatively normal distribution. Further, correlation results were examined for each sample of the study, as seen in Table 3.

**Table 2:** Observed scale characteristics.

		**Australia**	**China**	**Iran-Pre 5th COVID-19 waves**	**Iran-Post 5th COVID-19 waves**	**Turkey**
Parent attitudes about child vaccines	*Mean*	10.34	9.82	11.69	11.41	10.03
	*SD*	3.54	311	3.00	3.18	3.34
	*Max/Min*	4/20	4/20	4/20	4/20	4/20
	Skewness	−0.32	−0.32	−0.90	−0.83	−0.39
	Kurtosis	−0.99	−0.81	0.23	−0.12	−0.68
	Internal reliability	0.87	0.87	0.76	0.80	0.84
Child vulnerability	*Mean*	10.14	15.07	11.05	11.01	9.97
	*SD*	3.85	3.87	3.86	3.77	3.35
	*Max/Min*	8/40	8/40	8/40	8/40	8/40
	Skewness	0.62	0.35	0.55	0.49	0.70
	Kurtosis	0.07	0.50	0.13	−0.02	1.02
	Internal reliability	0.81	0.81	0.80	0.79	0.81
Financial well-being	*Mean*	14.90	12.69	13.32	13.65	13.77
	*SD*	6.25	3.84	3.88	3.69	3.81
	*Max/Min*	5/25	5/25	5/25	5/25	5/25
	Skewness	−0.73	−0.16	−0.42	−0.51	−0.66
	Kurtosis	0.05	−0.98	−0.43	0.12	−0.06
	Internal reliability	0.85	0.87	0.73	0.74	0.84
Parental vaccine hesitancy	*Mean*	24.16	12.87	24.38	24.65	23.74
	*SD*	6.25	3.89	3.89	3.80	4.42
	*Max/Min*	10/50	10/50	10/50	10/50	10/50
	Skewness	−1.33	0.45	−0.56	−0.55	−1.10
	Kurtosis	0.99	0.15	0.33	0.19	2.32
	Internal reliability	0.81	0.76	0.81	0.84	0.70

**Table 3:** Correlation results for the study variables.

	**1**.	**2**.	**3**.	**4**.
**Australia (*****n** =* **2734)**				
1. Parent attitudes about child vaccines	–	0.06^*^	−0.12^**^	−0.58^**^
2. Child vulnerability		–	−0.26^**^	0.14^**^
3. Financial well-being			–	0.01
4. Parental vaccine hesitancy				–
**China (*****n** =* **523)**				
1. Parent attitudes about child vaccines	–	0.09^*^	0.12^**^	0.08
2. Child vulnerability		–	0.30^**^	0.18^**^
3. Financial well-being			–	−0.08^*^
4. Parental vaccine hesitancy				–
**Iran-Pre (*****n** =* **1187)**				
1. Parent attitudes about child vaccines	–	0.08^**^	−0.16^**^	−0.26^**^
2. Child vulnerability		–	−0.38^**^	−0.05
3. Financial well-being			–	0.10^**^
4. Parental vaccine hesitancy				–
**Iran-Post (*****n** =* **1260)**				
1. Parent attitudes about child vaccines	–	0.17^**^	−0.16^**^	−0.35^**^
2. Child vulnerability		–	−0.36^**^	−0.11^**^
3. Financial well-being			–	0.09^**^
4. Parental vaccine hesitancy				–
**Turkey (*****n** =* **369)**				
1. Parent attitudes about child vaccines	–	0.16^**^	−0.24^**^	−0.29^**^
2. Child vulnerability		–	−0.29^**^	−0.02
3. Financial well-being			–	0.02
4. Parental vaccine hesitancy				–

* *P* < 0.05,

** *P* < 0.001.

The measurement models were then examined for the latent variables included in the mediation model using confirmatory factor analysis. The results indicated poor-to-adequate data-model fit statistics for each measure included in the study; therefore, modification indices, factor loadings (i.e., regression weights), and residual variances were examined in terms of countries to improve the measurement models. After excluding low loading items, which had regression weights <0.40 (107, 108), the measurement models were rerun. The modified measurement models provided better data-model fit statistics, as shown in Table 4.

**Table 4:** Model fit statistics for the confirmatory factor analyses.

	***χ^2^***	***df***	**CFI**	**RMSEA (95%)**
**Australia (*****n** =* **2,734)**				
1. PACV	88.95	2^**^	0.99	0.12 (0.10,0.14)
2. CVS	3.37	2	0.99	0.02(0.00,0.04)
3. FWB	13.27	2^*^	0.99	0.04 (0.02,0.07)
4. PVH	14.03	2^*^	0.99	0.04 (0.03,0.07)
**China (*****n** =* **523)**				
1. PACV	6.14	2^*^	0.99	0.06 (0.07,0.12)
2. CVS	16.28	5^**^	0.96	0.11 (0.06,0.13)
3. FWB	0.12	2	0.99	0.00 (0.00,0.02)
4. PVH	0.86	2	0.99	0.00 (0.00,0.06)
**Iran-Pre (*****n** =* **1,187)**				
1. PACV	3.23	2	0.99	0.02 (0.00,0.06)
2. CVS	8.35	5^*^	0.99	0.05 (0.02,0.09)
3. FWB	37.50	2^**^	0.98	0.12 (0.09,0.15)
4. PVH	4.97	2	0.99	0.03 (0.00,0.07)
**Iran-Post (*****n** =* **1,260)**				
1. PACV	6.03	2^*^	0.99	0.04 (0.00,0.07)
2. CVS	4.69	5	0.99	0.03 (0.00,0.06)
3. FWB	23.98	2^**^	0.99	0.09 (0.06,0.12)
4. PVH	21.83	2^**^	0.98	0.08 (0.05,0.12)
**Turkey (*****n** =* **369)**				
1. PACV	3.62	2	0.99	0.05 (0.00,0.12)
2. CVS	9.55	5^*^	0.98	0.10 (0.04,0.11)
3. FWB	0.51	2	0.99	0.04 (0.00,0.06)
4. PVH	3.09	2	0.99	0.03 (0.00,0.11)

PACV, Parent attitudes about child vaccines; CVS, child vulnerability scale; FWB, financial well-being; PVH, Parental Vaccine Hesitancy.

* *P* < 0.05,

** *P* < 0.001.

Measurement invariance were established across countries for the latent variables included in the model (109). Measurement invariance was utilized to examine configural, metric, and scalar invariance for countries using multiple-groups confirmatory factor analysis. Findings from these analyses were interpreted utilizing the ΔCFI and ΔRMSEA scores, with scores < 0.01 accepted as evidence of invariance across counties (110). Results from multi-group analyses indicated that measurement models, which were comprised of configural, metric, and scalar invariance, provided good-data model fit statistics across countries, as seen in Table 5. Given the change in the values of the CFI (ΔCFI < 0.01), although measurement invariance was observed at the configural invariance for all measures, it was not observed at the metric and scalar invariance levels for the PACV, FWB, and PVH. Measurement invariance of the CVS was also observed at the configural, metric, and scalar level.

**Table 5:** Model fit statistics for the multi-group confirmatory factor analyses.

	***χ^2^***	***df***	**CFI**	**RMSEA (95%)**	**ΔCFI**	**ΔRMSEA**
**Measurement invariance model of the PACV**						
Configural	107.95	10^**^	0.991	0.040 (0.03,0.05)		
Metric	449.80	22^**^	0.962	0.057 (0.05,0.06)	0.029	−0.017
Scalar	561.66	26^**^	0.952	0.058 (0.05,0.06)	0.01	−0.001
**Measurement invariance model of the CVS**						
Configural	42.29	10^**^	0.994	0.023 (0.01,0.03)		
Metric	109.87	22^**^	0.985	0.026 (0.02,0.03)	0.009	0.003
Scalar	114.89	26^*^	0.985	0.024 (0.02,0.03)	0.000	−0.002
**Measurement invariance model of the FWB**						
Configural	75.37	10^**^	0.993	0.033 (0.02,0.04)		
Metric	301.52	22^**^	0.972	0.046 (0.04,0.05)	0.021	−0.013
Scalar	316.54	26^**^	0.971	0.043 (0.03,0.05)	0.001	0.003
**Measurement invariance model of the PVH**						
Configural	82.51	10^**^	0.995	0.035 (0.03,0.04)		
Metric	278.59	22^**^	0.982	0.044 (0.04,0.05)	0.013	−0.009
Scalar	1,066.95	26^*^	0.927	0.081 (0.07,0.08)	0.055	−0.037

* *P < 0.05*,

** *P < 0.001*,

Finally, the mediating role of parent attitudes toward vaccines and child vulnerability in the link between financial wellbeing and parental vaccine hesitancy was tested. The results of the proposed model provided good-data model fit statistics (χ^2^= 14.52, *df* = 5, *p* = 0.01, CFI = 0.99, TLI = 0.98, RMSEA [95% CI] = 0.02 [0.01, 0.03]). In Australia, standardized regression estimates revealed that financial well-being had significant and negative associations with parent attitudes about vaccines and child vulnerability, but was not a significant predictor of parental vaccine hesitancy. Parental vaccine hesitancy was also predicted by parent attitudes about vaccines and child vulnerability. The indirect link of financial well-being with parental vaccine hesitancy through parent attitudes about vaccines and child vulnerability is significant, as shown in Table 6.

**Table 6:** Model paths indicating the direct and indirect associations between the variables of the study.

	**Standardized effects**		**BC 95% CI**	
**Direct**	**Indirect**	**Lower**	**Upper**
**Australia (*****n** =* **2,734)**				
FWB-- → PACV	−0.09^**^			
FWB-- → CVS	−0.20^**^			
PACV-- → PVH	−0.67^**^	0.04^**^	0.02	0.06
CVS-- → PVH	0.11^**^			
FWB-- → PVH	−0.03			
**China (*****n** =* **523)**				
FWB-- → PACV	0.12^*^			
FWB-- → CVS	0.40^**^			
PACV-- → PVH	0.09^*^	0.04^*^	0.01	0.08
CVS-- → PVH	0.08^*^			
FWB-- → PVH	−0.13^*^			
**Iran-Pre (*****n** =* **1,187)**				
FWB-- → PACV	−0.16^**^			
FWB-- → CVS	−0.36^**^			
PACV-- → PVH	−0.30^**^	0.06^**^	0.04	0.09
CVS-- → PVH	−0.04			
FWB-- → PVH	−0.04			
**Iran-Post (*****n** =* **1,260)**				
FWB-- → PACV	−0.15^**^			
FWB-- → CVS	−0.33^**^			
PACV-- → PVH	−0.41^**^	0.08^**^	0.05	0.11
CVS-- → PVH	−0.06^*^			
FWB-- → PVH	0.00			
**Turkey (*****n** =* **369)**				
FWB-- → PACV	−0.19^**^			
FWB-- → CVS	−0.20^**^			
PACV-- → PVH	−0.31^**^	0.07^**^	0.04	0.11
CVS-- → PVH	−0.05			
FWB-- → PVH	−0.04			

BC 95% CI for standardized indirect effects: bootstrapped bias-corrected and accelerated confidence interval with sample 5000.

* *P < 0.05*,

** *P < 0.001*,

The results from Chinese participants indicated that financial well-being had significant and positive predictive effects on parent attitudes about vaccines, child vulnerability, and parental vaccine hesitancy. Additionally, parental vaccine hesitancy was predicted by parent attitudes about vaccines and child vulnerability, and these variables mediated the association between financial well-being and parental vaccine hesitancy. The model was then examined with Iranian participants. In the first sample, the model showed that financial well-being had significant and negative associations with parent attitudes about vaccines and child vulnerability, but was not a significant predictor of parental vaccine hesitancy. Although parental vaccine hesitancy was significantly predicted by parent attitudes about vaccines, it did not predict child vulnerability. The indirect link of financial well-being with parental vaccine hesitancy through parent attitudes about vaccines is significant.

The results of the second Iranian sample revealed that financial well-being had significant and negative associations with parent attitudes about vaccines and child vulnerability, but was not a significant predictor of parental vaccine hesitancy. Parent attitudes about vaccines and child vulnerability also significantly and negatively predicted parental vaccine hesitancy. Financial well-being had a significant association with parental vaccine hesitancy through parent attitudes about vaccines and child vulnerability.

Turkish parents' financial well-being had significant and negative relationships with parent attitudes about vaccines and child vulnerability; however, it did not significantly predict parental vaccine hesitancy. Parental vaccine hesitancy, on the other hand, then was significantly predicted by parent attitudes about vaccines. The indirect link of financial well-being with parental vaccine hesitancy through parent attitudes about vaccines is significant, as seen in Table 6.

## Discussion

In the present study, we aimed to investigate the mediating role of parent attitudes toward COVID-19 vaccines and child vulnerability in the link between perceived financial well-being and parental vaccine hesitancy.

The current study indicated the mediating role of parent attitudes toward vaccines and child vulnerability in the link between financial well-being and parental vaccine hesitancy as a model in four countries including Australia, China, Iran, and Turkey. The COVID-19 vaccine hesitancy among parents is a worldwide health concern that has been investigated in different countries (17, 83). A wide range of factors that influence parents' vaccine hesitancy have been identified including ethnicity, family income, type of insurance, social media use (83), and uncertainty about vaccines (17, 111). It has been suggested that vaccine safety and effectiveness are two main concerns regarding the COVID-19 vaccine (26, 112). The current study support the findings from previous research. Studies have indicated that an individual's financial comfort can be considered as a predictor factor of vaccine hesitancy (113). Furthermore, attitudes toward vaccine safety and effectiveness predict the willingness of parents to get their children vaccinated against COVID-19 (114). The current study indicated the mediating role of parents' perception of their child's vulnerability in the relationship between financial well-being and vaccine hesitancy. This means that the degree to which the parents perceive their child as vulnerable to infection by COVID-19 can predict their willingness to get their children vaccinated regardless of their financial status. The theory of protection motivation (PMT) can help to explain the current findings. Accordingly, individual fear appraisal can make attitudes change (91). When individuals perceive the susceptibility and the severity of a situation, their knowledge, attitude, and performance may change (115, 116).

The current study revealed that financial well-being had significant and negative associations with parents' attitudes toward the COVID-19 vaccine and child vulnerability among the Australian participants. Generally, Australia has a high vaccine uptake in comparison with other high-income countries such as the US and Canada (117). However, some studies have indicated public concerns over the safety of existing COVID-19 vaccines (118, 119) that can raise parents' concerns regarding the safety and effectiveness of the vaccine for their children. Studies have determined several factors affecting parents' decision on childhood vaccination in Australia that include concerns such as potential side effects and vaccine safety (120). The current findings related to perceived financial well-being and parental vaccine hesitancy in Australia can be explained through past research. For instance, Swaney and Burns (86) found that Australian parents with self-reported higher-socioeconomic status were more likely to be vaccine-hesitant because they perceived themselves as educated and not wanting to control their children's health decisions. Also, they believed their families were safe from disease and vaccines posed a greater risk. Furthermore, they reported a belief that their lifestyle factors can protect them from vaccine-preventable diseases (86).

This study also revealed that parental vaccine hesitancy was predicted by Australian parents' attitudes toward the COVID-19 vaccine and perception of their child's level of vulnerability. One study (121) indicated that although parents expressed a strong desire for protecting their children, almost half of parents did not intend to vaccinate their children because they had concerns about the vaccine's long-term effects on child health and development.

Contrary to the Australian findings, the results of the Chinese participants indicated that perceived financial well-being had significant and positive predictive effects on parent attitudes toward vaccines, child vulnerability, and parental vaccine hesitancy. The findings of a population-based study (*n* = 2,463) indicated that more than 50% of Chinese parents were hesitant about the COVID-19 vaccine. While mothers were more hesitant, factors like the child's age (under 18 years old), knowledge deficit regarding the COVID-19 vaccinations, and lower awareness of the permission of vaccinating children were the determinants of parental vaccine hesitancy (77). The findings of Lu et al. (26) indicated that out of 3,673 parents more than 87.5% accepted the COVID-19 vaccine for their children. They believed that new vaccines, such as the COVID-19 vaccine, carry more risks than older vaccines. They also found that the parents' income was significantly related to vaccine hesitancy among Chinese parents. Parents with less than average income had lower hesitancy. This finding is supported by the result of the current study which showed that families with higher well-being perception have negative attitude toward the COVID-19 vaccine. The current study also revealed that financial well-being can predict Chinese parents' perceptions of their children's vulnerability. In general, children were considered as a vulnerable group and (122) living in a low-income family makes a child more vulnerable during the COVID-19 pandemic. The findings of a large survey (*n* = 20,632) conducted in China indicated that individuals with higher socioeconomic status worried less about COVID-19 as they had better education, higher income, and more resources in coping with COVID-19 (123) and therefore, may experience less stress. Published studies have addressed parental stress during the COVID-19 pandemic due to sociodemographic factors (124), their mental health (125), the children's distance education (126), the child's health status (127), and a variety of different factors. Contrary to this, the current study showed that Chinese parents who had the highest level of socioeconomic status perceived their children as more vulnerable to COVID-19.

Data from the Iranian participants revealed that parent' attitudes toward vaccines and child vulnerability significantly and negatively predicted parental vaccine hesitancy. Also, the study indicated that financial well-being had a significant association with parental vaccine hesitancy through parent attitudes about vaccines and child vulnerability. Some studies have investigated Iranian population's COVID-19 vaccination intent and have reported the importance of the role of trust in the healthcare systems (28), believing in COVID-19 vaccine effectiveness, low concern about vaccine safety, greater exposure to cues to vaccinate (128), as well as attitudes and subjective norms about the importance of COVID-19 vaccination, and using social media (129). Furthermore, Iranian intent to get COVID-19 vaccinated has been predicted by their attitudes, perceived COVID-19 infectability, and perceived behavioral control (130). Studies addressed that Iranian parents experienced fear during the COVID-19 outbreak and perceived their children as susceptible to infection (131). The children's vulnerability during the COVID-19 pandemic and following protective behavior was highlighted if the child had a chronic disease (132) or when individuals experienced economic issues (133). Iran has experienced more than five COVID-19 waves to date, and for children under 18 years old, vaccinations have only recently begun in Iran.

The current study revealed that financial well-being had significant and negative relationships with parent attitudes about vaccines and child vulnerability. However, it did not significantly predict parental vaccine hesitancy among Turkish parents. Parental vaccine hesitancy was significantly predictive of parents' attitudes about vaccines, but it did not predict the child's vulnerability. The findings of Ikiisik et al. (99) indicated that almost 90% of parents were hesitant about vaccinating their children with the COVID-19 vaccines. Age and risk perception were the identified factors that influence vaccine hesitancy. Another study indicated that only 36.3% of Turkish parents were willing to have their children receive the COVID-19 vaccine. Advising others to receive the vaccine was a significant predictor of parents' willingness to get the COVID-19 vaccine for their children (78). The correlation between attitude toward the COVID-19 vaccine and vaccine acceptance has been identified among the Turkish population (114). Kilic et al. (134) indicated that increasing fear of the COVID-19 contagion, having relatives infected with COVID-19, increasing perceived health status and life satisfaction, older age, being a male and not being a worker-tradesman increase the probability of having a positive attitude toward COVID-19 vaccine. Another study found that anxiety about the vaccine side effects, uncertainty of the vaccine effectiveness, and distrust of vaccines originating from abroad influence parental COVID-19 vaccine hesitancy (111).

Parental vaccine hesitancy, may also be predicted by the vaccine type and origin (75, 135). Parents may prefer conventional vaccines over mRNA vaccines due to lack of confidence in the mRNA technology and fear of its unknown side effects. Another concern among parents is the possible existence of microchips in some COVID-19 vaccines (135). Some studies showed that the general population trust COVID-19 vaccines manufactured by their government (75) while others may trust vaccines produced by the international, well-known, and specialized drug and vaccine companies. In general, vaccine acceptance is dynamic and time-dependent, and it can be influenced by different potential factors such as vaccine effectiveness, trust, vaccine safety, information, vaccine mandate, and fear (136).

## Study limitations

One of the limitations of this study is the use of online data gathering wherein only parents with access to a smartphone or other digital device were not included in the study. Hence, the findings of the current study may not be generalized. Furthermore, the Iranian data were gathered before and during the 5th pandemic wave and now Iran is in its 6th COVID-19 pandemic wave, therefore some findings may not be current. Additionally, the data were gathered between 8 August 2021 and 1 October 2021 and considering the nature of the COVID-19 pandemic and the occurrence of subsequent waves of the disease, the current study variables may have been affected. The COVID-19 vaccine has now been used for a large number of children, hence the phenomenon of parental COVID-19 vaccine hesitancy may have also changed. It is therefore recommended that more studies be conducted to investigate the validity of the proposed model.

## Conclusion

Children around the world are considered as a vulnerable group as their health is dependent on parents' or guardians' decisions. With the existence of the COVID-19 virus, vaccination of children is one of the best ways to protect them from the virus and prevent further spread of the disease. Despite the fact that COVID-19 vaccines are considered safe, vaccine hesitancy is common among parents. Vaccine hesitancy in the COVID-19 era is imbued in social, cultural, and historical contexts (137). The current study revealed that parental COVID-19 vaccine hesitancy could be predicted by parental perceptions of their child's vulnerability and their attitudes toward vaccines. Additionally, this study addressed the relationship between financial well-being and vaccine hesitancy, and suggests that consideration should be given to the type of vaccine messaging directed toward parents of varying economic groups regardless of what country they are located. Assessing the parents' vaccine hesitancy is recommended in public education campaigns to promote COVID-19 vaccination for children. Although the hospitalization and mortality rate of children due to COVID-19 infection has not been reported highly in various studies, COVID-19 infection among unvaccinated children can lead to more serious health consequences. In addition, contracting COVID-19 can deprive children of attending school and subsequently cause parents to be absent from work. On a macro scale, this would have adverse impact on the macro-economy.

## Data availability statement

The raw data supporting the conclusions of this article will be made available by the authors, without undue reservation.

## Ethics statement

This study was reviewed and approved by the Ethics Committee of Mazandaran University of Medical Sciences, Iran. Reference No: IR.MAZUMS.REC.1400.189. The participants provided their written informed consent to participate in this study.

## Author contributions

Study conception and design: HS and FK. Data collection: FK, HS, K-AA, LS, and OG. Analysis and interpretation of results: GA and HS. Draft manuscript preparation: FK, HK, K-AA, and ES. All authors reviewed the results and approved the final version of the manuscript.
